# Thriving on challenge stressors? Exploring time pressure and learning demands as antecedents of thriving at work

**DOI:** 10.1002/job.2115

**Published:** 2016-06-30

**Authors:** Roman Prem, Sandra Ohly, Bettina Kubicek, Christian Korunka

**Affiliations:** ^1^ University of ViennaViennaAustria; ^2^ University of KasselKasselGermany

**Keywords:** time pressure, learning demands, cognitive appraisal, thriving, diary study

## Abstract

In the conceptualization of thriving at work, it is emphasized that employees' learning and vitality are two equally important components of thriving and that thriving is facilitated by contextual features and available resources. In this study, we examined the effects of two challenge stressors (time pressure and learning demands) on thriving at work. Based on the literature on challenge and hindrance stressors, we proposed that challenge stressors positively affect learning and negatively affect vitality. To uncover underlying mechanisms, we measured challenge appraisal and hindrance appraisal of work situations in a diary study. A sample of 124 knowledge workers responded to three daily surveys (before the lunch break, during the afternoon, and at the end of the workday) for a period of five workdays. Results indicate that the indirect effects of learning demands and time pressure on learning are mediated by challenge appraisal, whereas indirect effects of learning demands on vitality are mediated by hindrance appraisal. Overall, our study shows that challenge stressors have a positive total effect on learning but no total effect on vitality. These differential relationships call for a finer distinction between the two components of thriving at work in future research. Copyright © 2016 The Authors Journal of Organizational Behavior Published by John Wiley & Sons Ltd.

We live in a fast‐paced world characterized by turbulent economic changes (e.g., Cascio, 1995; Rosa, 2003, 2013). In globalized markets, companies have to adapt quickly to changing environments to stay competitive. In order to be sustainable, it is becoming increasingly important for companies to maintain a thriving workforce energized to grow and develop (Spreitzer, Porath, & Gibson, 2012). Thriving at work can be described as a “psychological state in which individuals experience both a sense of vitality and a sense of learning at work” (Spreitzer, Sutcliffe, Dutton, Sonenshein, & Grant, 2005, p. 538). Thriving employees supposedly create new resources, such as knowledge, meaning, and strong social relationships, while performing their work. Consequently, thriving at work contributes to performance while improving employees' health at the same time (e.g., Porath, Spreitzer, Gibson, & Garnett, 2012).

In their socially embedded model of thriving at work, Spreitzer et al. (2005) highlight that learning and vitality are two equally important components of thriving. They further propose that contextual features and available resources positively affect both learning and vitality at work (Spreitzer et al., 2005). The assumption that available resources affect both components has been supported for positive meaning at work, as the experience of positive meaning in the morning increases feelings of learning and vitality at the end of the workday (Niessen, Sonnentag, & Sach, 2012). However, although available resources affect both learning and vitality in the same way, it is questionable whether this would also hold for other work characteristics. Thus far, scholars have not considered that certain job stressors might also hold the potential to foster thriving at work.

In the literature, job stressors are differentiated into challenge stressors and hindrance stressors (Cavanaugh, Boswell, Roehling, & Boudreau, 2000; Crawford, LePine, & Rich, 2010; LePine, Podsakoff, & LePine, 2005). Meta‐analytical evidence showed that although both types of job stressors have adverse effects, challenge stressors (as opposed to hindrance stressors) also have favorable effects, for example, on motivation and performance (Crawford et al., 2010; LePine et al., 2005; Podsakoff, LePine, & LePine, 2007). Thus, we propose that exposure to challenge stressors might hold the potential for both positive and negative effects on employees' thriving at work.

Because coping with challenge stressors may be motivating and provide opportunities for personal growth and learning at work (LePine et al., 2005; Paulsson, Ivergård, & Hunt, 2005), we propose that challenge stressors positively affect employees' learning at work. At the same time, coping with challenge stressors can also be effortful and increase strain (e.g., Bakker & Demerouti, 2007), and therefore, we propose depleting effects on employees' vitality at work. Overall, there seems to be reason to believe that challenge stressors will have differential effects on the two components of thriving at work.

In this paper, we pursue two goals: First, we examine possible differential effects of two challenge stressors (time pressure and learning demands) on learning and vitality at work. To our knowledge, this is the first study examining whether, aside from available resources, challenge stressors might also positively influence thriving at work. It is also the first study to examine differential effects on the two components of thriving at work: learning and vitality. Second, as we propose cognitive appraisal processes to play an important role as underlying mechanisms explaining the relationships between challenge stressors and both components of thriving at work, we measure employees' challenge and hindrance appraisal of their work situation in a diary study. Asking participants about their cognitive appraisals directly on multiple workdays enables us to get a more accurate understanding of the underlying within‐person processes.

## The concept of thriving at work

In recent years, the concept of thriving at work has emerged and received a great deal of attention in positive organizational scholarship (e.g., Paterson, Luthans, & Jeung, 2014; Spreitzer & Porath, 2012). Thriving at work is characterized by the joint experience of learning and vitality (Spreitzer & Sutcliffe, 2007; Spreitzer et al., 2005). When employees experience learning, they have a sense that they are continually improving and getting better at what they do (Porath et al., 2012). The experience of vitality is characterized by positive feelings based on available energy and feelings of aliveness (Porath et al., 2012). Based on hedonic and eudaimonic perspectives on psychological functioning and development (cf. Ryan & Deci, 2001), Spreitzer et al. (2005) emphasize that only the joint experience of both cognitive (learning) and affective (vitality) components of thriving gives employees a sense that they are progressing in their self‐development.

The assumption that experiences of learning and vitality are equally important components of thriving at work distinguishes thriving from other concepts in positive organizational scholarship. For example, thriving and flourishing (e.g., Diener et al., 2010) share similarities, as they both involve positive states of human functioning. However, flourishing only requires the experience of either psychological or social well‐being and does not depend on experiences of learning (Spreitzer et al., 2005). Similarly, thriving at work and work engagement (e.g., Bakker, Schaufeli, Leiter, & Taris, 2008) share a conceptual overlap to some degree. In both concepts, available energy (termed vitality or vigor, respectively) is a main component. Still, work engagement does not require experiences of learning, as it is more strongly concentrated around experiences of dedication and absorption (Spreitzer, Lam, & Fritz, 2010).

Thriving at work provides benefits, not only for the employees themselves but also for the organizations in which they work. Porath et al. (2012) have shown that thriving at work is related to better general health and well‐being, less strain, and lower levels of burnout. Further, thriving at work is positively associated with career development initiative and thriving outside of work. In addition to these positive effects for the individual, thriving at work also leads to greater proactivity and better job performance (Paterson et al., 2014; Porath et al., 2012). Other positive effects of a thriving workforce for organizations are higher levels of innovative work behavior (Carmeli & Spreitzer, 2009) and increased innovation (Wallace, Butts, Johnson, Stevens, & Smith, 2016).

In their socially embedded model of thriving at work, Spreitzer et al. (2005) also emphasized the importance of contextual features and resources as antecedents of thriving at work. It has been proposed that the contextual features of the work unit (e.g., decision‐making discretion and a climate of trust and respect) and resources that are produced in work processes (e.g., positive meaning and knowledge) promote experiences of learning and vitality through more agentic work behaviors. Initial empirical findings supported these assumptions (e.g., Niessen et al., 2012; Paterson et al., 2014). In a diary study over five workdays, Niessen et al. (2012) showed that perceived resources in the morning positively affect employees' learning and vitality at the end of the workday through more agentic work behaviors. The diary study by Niessen et al. (2012) further showed that both components of thriving—learning and vitality—have meaningful fluctuations on the day level and that available resources indeed affect thriving at work, even on very short timescales such as the day level.

## Challenge stressors as antecedents of thriving at work

Although the positive effects of available resources on thriving at work have already been investigated, the role of job stressors for employees' thriving has not received the same amount of attention. Spreitzer et al. (2005) “assume that the contextual enablers of thriving are not merely the opposite factors that exacerbate stress” (p. 539) and that “thriving is not cultivated simply by decreasing stressors” (p. 539). This assumption implicitly suggests that job stressors have negligible or negative effects on employees' thriving at work. To our knowledge, Spreitzer and colleagues (e.g., Spreitzer & Sutcliffe, 2007; Spreitzer et al., 2005) never explicitly ruled out that some job stressors could be potential facilitators of thriving at work. Nevertheless, because job stressors have not been considered antecedents of thriving at work in previous theorizing, the role of job stressors in relation to employees' learning and vitality at work is an open question.

It makes sense that many stressors do not have any favorable effects on learning and vitality. Nevertheless, it also seems plausible to assume that at least some stressors foster employees' thriving at work. Studies based on the literature on challenge and hindrance stressors (Cavanaugh et al., 2000) have shown that certain so‐called *challenge stressors* are positively related to motivation and performance (LePine et al., 2005), commitment (Podsakoff et al., 2007), and engagement (Crawford et al., 2010). Besides these positive effects, challenge stressors are also known to promote strain (e.g., LePine et al., 2005). Thus, challenge stressors have differential effects on work outcomes (Widmer, Semmer, Kälin, Jacobshagen, & Meier, 2012).

Although previous studies have found homologous effects of available resources on both components of thriving at work (e.g., Niessen et al., 2012) and both components are positively correlated (Porath et al., 2012), we do not expect challenge stressors to have homologous effects on learning and vitality at work. Because of their ambivalent nature (e.g., LePine et al., 2005; Widmer et al., 2012), we propose that challenge stressors also have differential effects on the two components of thriving at work.

In this paper, we focus on two challenge stressors: time pressure and learning demands. Time pressure has been defined as the extent to which employees feel that they need to work at a pace faster than usual or have insufficient time to finish their work tasks (Baer & Oldham, 2006; Kinicki & Vecchio, 1994). Learning demands require employees to acquire knowledge and skills that are necessary to perform their jobs effectively (Kubicek, Paškvan, & Korunka, 2015; Loon & Casimir, 2008). Empirical evidence showing that the prevalences of both time pressure and learning demands have increased over the last decades (e.g., Eurofound, 2012, 2015; Kubicek et al., 2015) underlines their growing importance in today's work settings. Moreover, time pressure and learning demands both represent typical challenge stressors in the sense that differential effects on work outcomes have been shown in previous studies (for time pressure, refer to Searle & Auton, 2015; Widmer et al., 2012; for learning demands, refer to Höge & Hornung, 2015; Paulsson et al., 2005).

Based on these research results, we expect that time pressure and learning demands are differentially related to the two components of thriving at work. Specifically, we propose positive effects of both challenge stressors on learning, as they potentially provide opportunities for personal growth. In the literature on challenge and hindrance stressors, it is argued that employees who cope successfully with time pressure will experience a sense of personal accomplishment (LePine et al., 2005, p. 765). Thus, time pressure should positively affect the learning component of thriving at work. Further, when coping with learning demands, the acquired knowledge and skills should provide employees with a sense of personal growth and learning at work. Thus, learning demands should also positively affect the learning component of thriving at work.

In contrast, we propose negative effects of both challenge stressors on vitality, as they provoke strain and deplete employees' energy throughout the workday. To meet performance requirements when coping with time pressure, employees have to invest additional effort in their work (Hockey, 1997). For example, employees have to resist distractions and concentrate on their tasks to meet their work goals when working to tight deadlines. Similarly, coping with learning demands may also require an investment of effort. For example, when familiarizing themselves with new work processes, employees may have to overcome inner resistances and persist in trying after initial setbacks. Because effort to resist distractions and overcome inner resistances have been shown to draw on employees' self‐regulatory resources (e.g., Prem, Kubicek, Diestel, & Korunka, 2016; Rivkin, Diestel, & Schmidt, 2015), we hypothesize negative effects of both time pressure and learning demands on employees' vitality.

Based on our assumptions about the differential effects of challenge stressors, we will not investigate the effects of time pressure and learning demands on thriving overall but explore their effects on learning and vitality on the day level. We propose

Hypothesis 1 Challenge stressors, i.e., (a) time pressure and (b) learning demands, experienced in the morning have a positive total effect on learning at the end of the workday.

Hypothesis 2 Challenge stressors, i.e., (a) time pressure and (b) learning demands, experienced in the morning have a negative total effect on vitality at the end of the workday.

## Challenge appraisal and hindrance appraisal as explanatory mechanisms

Recent advancements within the literature on challenge and hindrance stressors emphasize the importance of measuring cognitive appraisals directly from research participants (e.g., Webster, Beehr, & Love, 2011). Earlier researchers implicitly assumed, based on the transactional theory of stress (Lazarus & Folkman, 1984), that cognitive appraisals should be able to explain positive and negative effects of job stressors (e.g., LePine et al., 2005). Still, most studies relied on *a priori* classifications of stressors as challenge stressors or hindrance stressors either by the researchers or by way of ratings from different samples (e.g., Cavanaugh et al., 2000; Crawford et al., 2010; LePine et al., 2005).

Empirical research measuring cognitive appraisals directly from research participants showed that cognitive appraisals are able to explain the effects of job stressors on work outcomes (e.g., Webster et al., 2011). In diary studies, it has been shown that employees exposed to challenge stressors appraise their work situation as both more challenging and more hindering simultaneously (e.g., Searle & Auton, 2015). Further, both challenge appraisal and hindrance appraisal were shown to have meaningful fluctuations within persons on the day level that explain the effects of job stressors on proactivity and creativity (Ohly & Fritz, 2010) as well as positive affect and anger (Searle & Auton, 2015).

Challenge appraisal reflects an individual's perception of a situation as having potential for growth, mastery, or gain (Folkman & Lazarus, 1985), whereas hindrance appraisal can be described as an individual's frustration because of being obstructed in pursuing self‐relevant goals (Searle & Auton, 2015). Although challenge appraisal and hindrance appraisal might seem mutually exclusive, empirical data show that they are not (e.g., Searle & Auton, 2015). This could be explained by some aspects of a situation being perceived as having potential for growth, mastery, or gain, whereas other aspects of the same situation are perceived as an obstruction in the pursuit of self‐relevant goals. Thus, we argue that time pressure and learning demands will be positively related to both challenge appraisal and hindrance appraisal.

Effective coping with time pressure and learning demands often results in feelings of accomplishment and personal growth (e.g., LePine et al., 2005). Thus, in a work situation characterized by high levels of time pressure and/or learning demands, employees will likely perceive their work situation as having potential for growth, mastery, or gain. In other words, employees will appraise their work situation as challenging. At the same time, employees facing time pressure and/or learning demands might have to invest their time and attention to be able to meet a specific deadline, or, to acquire the necessary knowledge and skills. This could obstruct them from pursuing self‐relevant work goals, such as working on a less urgent project to which they feel more committed. Thus, employees might also appraise their work situation as hindering.

We hypothesize that the challenge appraisal of the work situation will have positive effects on thriving at work. It has been argued that cognitive appraisals affect work outcomes via their effects on motivation, affect, and coping behaviors. Challenge appraisal should increase motivation (LePine et al., 2005), positive affect (LePine, LePine, & Saul, 2007; Searle & Auton, 2015), as well as problem‐focused coping behavior (LePine et al., 2005; Searle & Auton, 2015). Because challenge appraisals lead to increased motivation and more positive affect, employees' available energy (Shirom, 2011) and vitality should be boosted. Further, more problem‐focused coping behavior as well as a broader thought–action repertoire promoted by higher positive affect (Fredrickson, 2001) should lead to more creativity and development and ultimately more learning at work. By combining our assumptions that challenge stressors, such as time pressure and learning demands, promote challenge appraisal and that challenge appraisal promotes vitality and learning at work, we propose the following indirect effects:

Hypothesis 3 Challenge stressors, i.e., (a) time pressure and (b) learning demands, experienced in the morning have a positive indirect effect on learning at the end of the workday via day‐level challenge appraisal of the work situation.

Hypothesis 4 Challenge stressors, i.e., (a) time pressure and (b) learning demands, experienced in the morning have a positive indirect effect on vitality at the end of the workday via day‐level challenge appraisal of the work situation.

We hypothesize that hindrance appraisals of the work situation will have negative effects on thriving at work. When employees are obstructed from pursuing self‐relevant work goals, this could lead to tendencies to withdraw from the situation or to show more aggressive behavior (Mackey & Perrewé, 2014; Perrewé & Zellars, 1999). As both withdrawal and aggression are inappropriate in work settings, employees will likely have to overcome their tendencies for these behaviors by investing their available energy in self‐regulation (Prem et al., 2016; Rivkin et al., 2015; Schmidt & Neubach, 2007). Consequently, hindrance appraisals should have adverse effects on employees' vitality. Further, hindrance appraisals promote more emotion‐focused coping behavior (LePine et al., 2005; Mackey & Perrewé, 2014) and increase negative affect (Searle & Auton, 2015). When employees focus their coping behavior more strongly on their emotions, we argue that they will be less likely to attain feelings of accomplishment and growth. Increased negative affect should further impede creativity and development because it narrows employees' thought–action repertoire (Fredrickson, 2001). Overall, hindrance appraisals should also have negative effects on employees' learning at work. By combining our assumptions that challenge stressors, such as time pressure and learning demands, promote not only challenge appraisal but also hindrance appraisal and that hindrance appraisal thwarts vitality and learning at work, we propose the following indirect effects:

Hypothesis 5 Challenge stressors, i.e., (a) time pressure and (b) learning demands, experienced in the morning have a negative indirect effect on learning at the end of the workday via day‐level hindrance appraisal of the work situation.

Hypothesis 6 Challenge stressors, i.e., (a) time pressure and (b) learning demands, experienced in the morning have a negative indirect effect on vitality at the end of the workday via day‐level hindrance appraisal of the work situation.

## Conceptual model of the study

It may seem that some of our previously mentioned hypotheses contradict one another. For example, hypotheses 1 and 3 predict positive total and indirect effects of challenge stressors on learning, whereas hypothesis 5 predicts negative indirect effects of challenge stressors on learning. Similarly, hypotheses 2 and 6 predict negative total and indirect effects of challenge stressors on vitality, whereas hypothesis 4 predict positive indirect effects of challenge stressors on vitality. These apparent conflicts can be resolved when considering that the total effect of a predictor on an outcome is the sum of all indirect effects and the remaining direct effect (e.g., Hayes, 2013, pp. 128–130).

**Figure 1:**
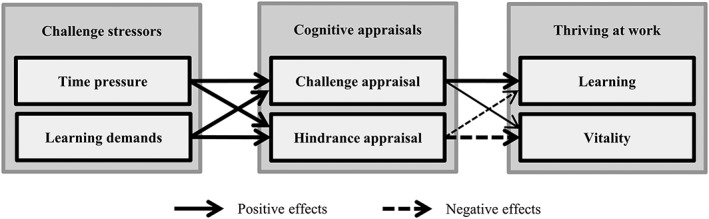
Conceptual model of the study

Implicit in the previously mentioned hypotheses rests the assumptions that the sizes of indirect effects will differ. As we hypothesize a positive total effect of challenge stressors on learning at work, positive indirect effects via challenge appraisal should be stronger than negative indirect effects via hindrance appraisal in the case of learning at work. In contrast, for vitality at work, the negative indirect effects of challenge stressors via hindrance appraisal should outweigh the positive indirect effects via challenge appraisal, resulting in the hypothesized negative total effect. As illustrated in Figure 1, we expect the pattern of total effects to arise because of the effects of challenge appraisal on learning being larger than the effects of hindrance appraisal on learning, and vice versa for the effects on vitality.

## Method

### Sample and procedure

As our study focused on within‐person effects of time pressure and learning demands, we decided to recruit knowledge workers to participate in a diary study (Fisher & To, 2012; Ohly, Sonnentag, Niessen, & Zapf, 2010). Knowledge work includes the creation of new knowledge, the application of existing knowledge to current problems, the teaching of knowledge, or the acquisition of existing knowledge through learning (Kelloway & Barling, 2000). Typical knowledge worker professions include scientists and professors, engineers (e.g., R&D workers, software designers, and telecommunication specialists), lawyers and attorneys, physicians, as well as financial analysts and accountants (Benson & Brown, 2007; Nomikos, 1989). We chose a sample of knowledge workers, as we expected time pressure and especially learning demands to have meaningful variation on the day level in knowledge work.

Participants were recruited by the first author and four undergraduate students through their private networks. All participation was voluntary. As an incentive for participation, we provided feedback about the study's results.

Participants were asked to complete a general survey and multiple daily surveys. The general survey had to be filled out before starting the daily surveys. Participants were instructed to complete the daily surveys three times a day on five consecutive workdays: (1) before the lunch break, (2) during the afternoon, and (3) directly after finishing work at the end of their workday.

The general survey was completed by 170 individuals, who provided a total of 647 day‐level datasets before the lunch break, a total of 571 day‐level datasets during the afternoon, and a total of 516 day‐level datasets after work. We matched day‐level datasets from the same individual and the same day if they were in the correct chronological order and consecutive entries were at least 30 min apart from one another. Matching resulted in 379 complete day‐level datasets from 124 individuals. We further excluded three day‐level datasets from one individual who kept providing diary entries after finishing the requested number of five workdays. Thus, the final dataset used in the analyses consisted of 376 days nested within 124 individuals.

In the final sample (52.4% female), the mean age was 35.5 years (*SD* = 10.6); mean job tenure was 7.7 years (*SD* = 8.6); mean working time was 41.9 h per week (*SD* = 9.6). As could have been expected, education levels were quite high in our sample of knowledge workers: 66.1% had a college degree, 29.9% had finished a college‐preparatory second education, and 4.0% had received a lower secondary education that in most cases was combined with vocational training. The final sample included professors and scientists (16.1%), engineers and IT personnel (12.9%), consultants (11.3%), teachers and educators (9.7%), managers and directors (9.7%), secretaries and assistants (8.1%), accountants and controllers (7.3%), specialists in human resources or marketing (6.5%), and other professions (18.5%, e.g., attorneys, journalists, physicians, psychologists).

### Measures

We used abbreviated scales for all measures to reduce participants' burden of filling out long scales multiple times (Fisher & To, 2012; Ohly et al., 2010). When abbreviating scales, we chose positively worded items only, because negatively worded items often impair psychometric properties of scales by loading on separate factors (Dalal & Carter, 2015). Further, we chose items that broadly cover the constructs being measured to maintain the scales' validity (Boyle, 1991; Fisher & To, 2012). Participants were instructed to answer all items with regard to their current workday, except for the vitality items, which participants answered with regard to their current well‐being. All items were administered in German on 5‐point scales (1 = *strongly disagree*; 5 = *strongly agree*).

*Time pressure* was assessed before the lunch break with three items adapted from the instrument for stress‐oriented job analysis (Semmer, Zapf, & Dunckel, 1999). A sample item is “This morning, I was pressed for time.”

*Learning demands* were measured before the lunch break with three items adapted from the intensification of job demands scale (Kubicek et al., 2015). A sample item is “This morning, I had to familiarize myself with new work processes.”

*Challenge appraisal* and *hindrance appraisal* of the work situation were measured during the afternoon with three items each adapted from Ohly and Fritz (2010). Sample items are “Today, I view my tasks as challenging.” and “Today, my work hinders me from attaining personal goals.”

*Learning* and *vitality* were measured at the end of the workday with three items each adapted from Porath et al. (2012). Sample items are “Today, I have developed as a person.” and “Right now, I feel alive and vital.”

As a control variable (cf. Additional analysis section), *positive meaning* was assessed before the lunch break with three items adapted from Spreitzer, Kizilos, and Nason (1997). A sample item is “This morning, the work I did was meaningful.”

To show that the variables measured in the daily diary represent empirically distinct constructs, we conducted multilevel confirmatory factor analyses (MCFAs) with Mplus 7.2 (Muthén & Muthén, 1998–2012). The MCFAs showed a satisfactory fit of the hypothesized seven‐factor model (*χ* ^2^ = 464.0, *df* = 336, RMSEA = 0.03, CFI = 0.97, TLI = 0.96, AIC = 18,879.1) that was superior to the best‐fitting six‐factor model with challenge appraisal and learning loading on the same factor (*χ* ^2^ = 571.7, *df* = 348, RMSEA = 0.04, CFI = 0.94, TLI = 0.93, AIC = 18,962.7; Δ*χ* ^2^ = 107.6, Δ*df* = 12, *p* < .001), as well as the three‐factor model with one factor per measurement occasion (*χ* ^2^ = 1819.2, *df* = 372, RMSEA = 0.10, CFI = 0.62, TLI = 0.58, AIC = 20,162.2; Δ*χ* ^2^ = 1355.1, Δ*df* = 36, *p* < .001), and the one‐factor model (*χ* ^2^ = 2901.0, *df* = 378, RMSEA = 0.13, CFI = 0.34, TLI = 0.27, AIC = 21,232.1; Δ*χ* ^2^ = 2437.0, Δ*df* = 42, *p* < .001).

### Data analysis

We tested all hypotheses simultaneously using multilevel structural equation modeling (MSEM) techniques in Mplus 7.2 (Muthén & Muthén, 1998–2012), as suggested by Preacher and colleagues (Preacher, Zhang, & Zyphur, 2011; Preacher, Zyphur, & Zhang, 2010; Zhang, Zyphur, & Preacher, 2009). This method allowed us to conduct analyses at multiple levels at the same time in a single analysis. By decomposing the variance of variables into their between‐person and within‐person components, MSEM accounts for the fact that relationships might be different on the between‐person and the within‐person levels. Thus, multilevel mediation analyses with MSEM are less prone to biases than other techniques of multilevel mediation analysis (Zhang et al., 2009).

## Results

### Preliminary analyses

Means, standard deviation, and zero‐order correlations between study variables are shown in Table 1. As indicators of internal consistency, mean Cronbach's alpha averaged across days of participation as well as McDonald's omega (cf. Bolger & Laurenceau, 2013; Dunn, Baguley, & Brunsden, 2014 were calculated (refer to columns 6 and 7 in Table 1). Although internal consistency was somewhat low for both cognitive appraisal scales, all factor loadings were in the expected direction and significant.

**Table 1:** Means, standard deviations, Cronbach's alphas, day‐level variance, and zero‐order correlations of study variables.

	*M* a	*SD* a	*M* b	*SD* b	*α* c	*ω* d	1‐ICC^e^	1	2	3	4	5	6	7
1. Time pressure	2.02	0.80	2.02	1.05	.91	.88	67%	—	**0.20**	0.10	**0.19**	0.11	**0.20**	−0.08
2. Learning demands	2.16	0.76	2.12	0.99	.80	.74	64%	0.21	—	**0.25**	**0.27**	**0.16**	**0.28**	−0.01
3. Positive Meaning	3.09	0.82	3.12	1.03	.91	.88	61%	0.20	0.09	—	**0.34**	0.04	**0.30**	0.02
4. Challenge appraisal	2.96	0.70	3.01	0.82	.67	.60	61%	**0.30**	**0.43**	**0.70**	—	**0.16**	**0.45**	0.01
5. Hindrance appraisal	1.57	0.60	1.57	0.69	.59	.59	59%	**0.40**	0.08	**−0**.05	0.16	—	0.06	**−0.19**
6. Learning	2.42	0.79	2.48	0.91	.88	.80	49%	0.10	**0.65**	**0.34**	**0.55**	0.11	—	**0.21**
7. Vitality	2.86	0.73	2.87	0.98	.93	.93	76%	**−0.34**	0.22	0.03	0.11	**−0.40**	0.18	—

*Note*: Correlations below the diagonal are person‐level correlations (*n* = 124). Correlations above the diagonal are day‐level correlations (*n* = 376). Numbers in bold indicate *p* < .05 for between‐person and within‐person correlations.

a Means and standard deviations at the person level.

b Means and standard deviations at the day level.

c Mean Cronbach's alphas averaged across days of participation.

d McDonald's omega index of within‐person measurement reliability from multilevel measurement models.

e 1‐ICC = percentage of variance at the day level; ICC = variance at person level / (variance at day level + variance at person level).

Before testing our hypotheses, we also examined the degree of within‐person and between‐person variation in our data. There was substantial within‐person variation, ranging between 49% (for learning) and 76% (for vitality), calling for a multilevel approach to data analysis (refer to column 8 in Table 1).

### Hypotheses testing

We tested all hypotheses simultaneously in a single MSEM. Because the distribution of indirect effects is skewed in most cases, we followed a suggestion by Preacher and Selig (2012) and used the unstandardized estimates and standard errors to construct Monte Carlo 95% confidence intervals around the within‐person indirect and total effects (shown in Table 2).

**Table 2:** Within‐person indirect and total effects from MSEM with Monte Carlo confidence intervals.

		Monte Carlo 95% CI	
	Estimate	LL	UL
Indirect effects			
Time pressure → challenge appraisal → learning	0.04*	0.01	0.08
Time pressure → challenge appraisal → vitality	0.01	−0.01	0.03
Time pressure → hindrance appraisal → learning	−0.00	−0.01	0.01
Time pressure → hindrance appraisal → vitality	−0.02	−0.05	0.01
Learning demands → challenge appraisal → learning	0.08*	0.04	0.13
Learning demands → challenge appraisal → vitality	0.01	−0.02	0.05
Learning demands → hindrance appraisal → learning	−0.01	−0.02	0.01
Learning demands → hindrance appraisal → vitality	−0.02*	−0.07	−0.00
Total effects			
Time pressure → learning	0.11*	0.02	0.20
Time pressure → vitality	−0.09	−0.21	0.04
Learning demands → learning	0.20*	0.10	0.31
Learning demands → vitality	0.01	−0.14	0.15

*Note*: The table shows unstandardized estimates.

CI = confidence interval; LL = lower limit; UL = upper limit.

* Significant at *α =* .05 level based on Monte Carlo 95% CI.

**Figure 2:**
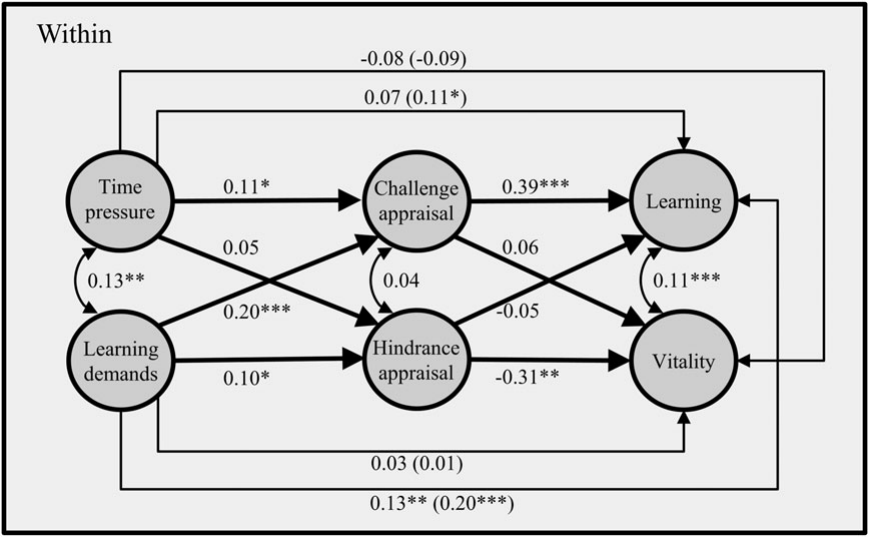
Within‐person estimates from multilevel structural equation modeling on outcomes; *p < .05, **p < .01, ***p < .001

Figure 2 illustrates the within‐person path estimates from the MSEM. As proposed, time pressure positively affected challenge appraisal. Although there was a marginally significant zero‐order correlation between time pressure and hindrance appraisal at the within‐person level (*r* = .11, *SE* = 0.06, *p* = .067), the effect of time pressure on hindrance appraisal was not significant in the MSEM when the effects of learning demands on hindrance appraisal were controlled for. Learning demands positively affected challenge appraisal as well as hindrance appraisal. We found unique patterns for the effects of both cognitive appraisals on the two outcomes. Each component of thriving was affected by only one specific type of cognitive appraisal. Challenge appraisal positively affected learning but had no effect on vitality, whereas hindrance appraisal had no effect on learning but was positively related to vitality.

Hypotheses 1a–b and 2a–b predicted total effects of time pressure and learning demands on the two outcomes: learning and vitality. To test these hypotheses, we calculated the total effects from the MSEM (shown in Table 2). The total effect of time pressure on learning was positive and significant. Although the total effect of time pressure on vitality was, as proposed, negative, it failed to reach significance (refer to Table 2). A similar pattern was found for the total effects of learning demands on learning and vitality. For learning demands, we found a positive and significant total effect on learning, but there was no total effect of learning demands on vitality (refer to Table 2). In summary, Hypotheses 1a and 1b, predicting positive total effects on learning, were supported, whereas Hypotheses 2a and 2b, predicting negative total effects on vitality, were not supported by our data.

Hypotheses 3a–b and 4a–b predicted indirect effects of time pressure and learning demands via challenge appraisal on the two outcomes: learning and vitality. As the confidence interval of the indirect effect of time pressure via challenge appraisal on learning did not include zero (refer to Table 2), Hypothesis 3a was supported. There were also indirect effects of learning demands via challenge appraisal on learning (refer to Table 2). Thus, Hypothesis 3b was supported as well. Because there was no effect of challenge appraisal on vitality, the respective indirect effects were also not significant (refer to Table 2). Consequently, Hypotheses 4a and 4b were not supported.

Hypotheses 5a–b and 6a–b predicted indirect effects of time pressure and learning demands via hindrance appraisal on the two outcomes: learning and vitality. As there was no effect of hindrance appraisal on learning, the respective indirect effects were also not significant (refer to Table 2). Consequently, Hypotheses 5a and 5b had to be rejected. Further, because there was no significant effect of time pressure on hindrance appraisal, the indirect effect of time pressure via hindrance appraisal on vitality was not significant either (refer to Table 2). Finally, our analyses revealed an indirect effect of learning demands via hindrance appraisal on vitality (refer to Table 2). Consequently, Hypothesis 6a was not supported, but Hypothesis 6b was supported.

### Additional analysis

We included positive meaning as a control variable in our study. In the literature on thriving at work, positive meaning is described as the purpose and significance inherent in work (Spreitzer et al., 2005, p. 543). Previous day‐level studies had shown that positive meaning affects both components of thriving (Niessen et al., 2012). Further, it had been argued that positive meaning helps employees to reappraise their work situations (Spreitzer et al., 2005, p. 544). Therefore, we assumed that positive meaning might affect not only our outcomes—learning and vitality—but also our mediators, challenge appraisal, and hindrance appraisal. Consequently, we controlled for positive meaning in an additional analysis.

When adding positive meaning as a third predictor to our model, results showed that positive meaning had a total effect on learning but did not affect the vitality component of thriving. Further, positive meaning affected challenge appraisal and showed a positive indirect effect via challenge appraisal on learning. The effect of positive meaning on hindrance appraisal and all other remaining indirect effects of positive meaning were not significant. Controlling for positive meaning as an additional predictor did not change the pattern and significance of results regarding our hypotheses.

## Discussion

Our diary study showed that time pressure and learning demands have differential effects on both components of thriving at work. Both challenge stressors positively affected the learning component of thriving, but neither of them showed significant effects on vitality. Further, our results showed that underlying mechanisms differ for each component of thriving at work. Challenge appraisals explained the indirect effects of both challenge stressors on learning, whereas hindrance appraisals seemed to play a role in the indirect effects of challenge stressors on vitality. Finally, our additional analysis showed that although positive meaning boosts challenge appraisals and affects learning, it did not affect the conclusions we draw regarding our hypotheses.

Our findings showed differential effects of two typical challenge stressors on thriving at work. As proposed, we found positive effects of time pressure and learning demands on learning at work. Based on the literature on challenge and hindrance stressors (e.g., LePine et al., 2005; Widmer et al., 2012), we further proposed negative effects of challenge stressors on vitality at work but failed to find significant effects. This means that on workdays with higher levels of time pressure or learning demands during the morning, employees felt that they got better and improved more at what they do at work than on workdays with lower levels of these challenge stressors. At the same time, the amount of energy employees had available at the end of their workday did not differ between days with higher versus lower levels of time pressure or learning demands. A possible explanation for not finding negative effects of challenge stressors on vitality could lie in the timing of the measurements in our study. Following a previous example in the literature (Niessen et al., 2012), we asked participants to indicate learning and vitality at the end of their workday. Over the course of the workday, time pressure and learning demands might not only have had negative effects on vitality by taxing employees' energy but could have had also positive effects by creating new motivational energy (LePine et al., 2005; Searle & Auton, 2015). It might be that vitality rises initially because of energizing effects of challenge stressors but becomes lower later during the day when the energizing effects of challenge stressors wear off. Consequently, potential positive and negative effects might have cancelled each other out, resulting in the overall non‐significant effects of challenge stressors on vitality at the end of the workday. In future research, such potentially delayed effects of challenge stressors should be investigated. Nevertheless, with positive effects on learning and no significant effects on vitality, challenge stressors still affected the two components in different ways. Consequently, we think that it might not always be the best decision to measure thriving at work as a compound of learning and vitality (e.g., as in Paterson et al., 2014). For some research questions, it may make sense to look at each of the two components of thriving separately. This may especially be the case when investigating thriving at work in day‐level studies or when looking at different effects of challenge stressors on thriving at work.

Our results also indicate that cognitive appraisals play a role in the underlying mechanisms that link challenge stressors to thriving at work. The results show that learning and vitality were each affected by one specific type of cognitive appraisal. Challenge appraisal played a role in the indirect effects of both challenge stressors on learning, but not in the indirect effects of both challenge stressors on vitality. This means that on workdays with higher levels of time pressure and learning demands, employees appraised their work situation as more challenging. These higher levels of challenge appraisal, in turn, promoted learning at work on these workdays. It seems that employees perceiving that their work situation can facilitate growth, mastery, or gain will approach their work more positively and will therefore—comparable to a self‐fulfilling prophecy—learn more during the workday than employees who do not expect their work situation to promote growth, mastery, or gain. The opposite way around, hindrance appraisal seemed to play a role in the indirect effects of challenge stressors on vitality, but not in the indirect effects of challenge stressors on learning. It should be noted that we found an indirect effect via hindrance appraisal on vitality solely for learning demands, not for time pressure. This means that employees also appraised their work situation as more of a hindrance on workdays when they had to deal with higher levels of learning demands. These higher levels of hindrance appraisal, in turn, depleted the amount of energy they had available at the end of these workdays. Our results further indicate that challenge appraisals played a stronger role in the motivating processes of challenge stressors that give employees a sense of learning, whereas hindrance appraisals were more closely tied to energy‐depleting processes that tap on employees' available energy. This is in line with previous studies finding positive effects of challenge stressors through increases in motivation combined with negative effects through increases in strain (e.g., LePine et al., 2005).

Finally, in an additional analysis, we also controlled for positive meaning in our day‐level study. As previous studies had shown that positive meaning affects both components of thriving at work (Niessen et al., 2012) and we expected that positive meaning might affect cognitive appraisal processes (Spreitzer et al., 2005, p. 544), we tested positive meaning as an additional predictor. The results showed that the effects of both challenge stressors via cognitive appraisal on thriving at work could not be explained by positive meaning, ruling out potential alternative explanations of our results. Thus, the conclusions we draw regarding our hypotheses are stable regardless of whether or not we control for positive meaning in our analyses.

### Limitations and implications for future research

We believe that the current day‐level study provides valuable insights into the differential effects of challenge stressors on thriving at work. However, our study also has some limitations that should be mentioned.

First, all measures were based on self‐reports, which may raise concerns about common method bias (Podsakoff, MacKenzie, & Podsakoff, 2012). However, it is unlikely that participants took the time to think about how various measured constructs should be related to one another when indicating their current state (Fisher & Noble, 2004). Furthermore, we separated the measurement of the predictors, mediators, and outcomes, as suggested by Podsakoff et al. (2012). To rule out further sources of common method bias, future research could try to obtain data from different sources and/or implement a full cross‐lagged design that also allows for the testing of reciprocal effects and the modeling of autocorrelations within workdays (cf. Maxwell, Cole, & Mitchell, 2011).

Second, it should be noted that the internal consistencies of our cognitive appraisal scales were somewhat low. Still, the factor loadings were all in the expected direction and significant, and the overall model fit of the MCFA was good. This indicates that all study constructs including both appraisal scales could be distinguished empirically. Moreover, one has to keep in mind that low internal consistencies result in inflated standard errors, making it more difficult for researchers to detect significant effects. Thus, low reliability may have led to an underestimation of potential effects. Consequently, we suggest that for better internal consistency of the cognitive appraisal measures, future studies should either use all items from Ohly and Fritz (2010)—instead of abbreviating the scales, as we did—or alternatively adopt the scales recently developed by Searle and Auton (2015).

Third, in our day‐level study on the effects of challenge stressors on thriving at work, we opted to focus on time pressure and learning demands, because their differential effects on work outcomes had previously been demonstrated. Nevertheless, by focusing on two specific challenge stressors, we did not test our assumptions about the effects of challenge stressors on thriving at work exhaustively. Future studies should also explore the roles of other challenge stressors (e.g., responsibility, skill demands, job complexity) as potential antecedents of thriving at work.

Finally, it should be noted that our decision to use a sample of knowledge workers also potentially limits the generalizability of our findings. Because it has been shown that nurses tend to appraise time pressure as a hindrance rather than as a challenge (Bakker & Sanz‐Vergel, 2013), one might assume differences in the underlying cognitive appraisal processes between knowledge work and human service work. Thus, although we could not show indirect effects of time pressure through hindrance appraisal in our study, results might be different in a sample of nurses. We recommend that future research tests the generalizability of our findings, either in service work in general or more specifically in particular professions, like nursing.

### Practical implications and conclusions

Our day‐level study showed that challenge stressors can have differential effects on employees' thriving at work. Although challenge stressors can promote a sense of learning, they lack positive (as well as negative) effects on employees' vitality at the end of the workday. Because thriving at work has been defined as the joint experience of “both a sense of vitality and a sense of learning at work” (Spreitzer et al., 2005, p. 538), one could argue that, in a way, challenge stressors do not facilitate thriving at work after all. As time pressure and learning demands have positive effects on only one of the two components of thriving, they do not promote thriving at work in a strict sense.

Finally, we want to add and emphasize that research on challenge stressors has documented their health‐impairing effects (e.g., Crawford et al., 2010; LePine et al., 2005). Because some evidence indicates that time pressure and learning demands tend to increase over time (e.g., Eurofound, 2012, 2015; Kubicek et al., 2015), practitioners should keep their potential beneficial as well as health‐impairing effects in mind. Keeping an eye on contextual features as well as the availability of resources will become increasingly important for organizations to maintain a thriving workforce in turbulent economic times.
